# Compare Analysis for the Nanotoxicity Effects of Different Amounts of Endocytic Iron Oxide Nanoparticles at Single Cell Level

**DOI:** 10.1371/journal.pone.0096550

**Published:** 2014-05-13

**Authors:** Chen-Yu Huang, Tzong-Rong Ger, Zung-Hang Wei, Mei-Feng Lai

**Affiliations:** 1 Department of Power Mechanical Engineering, National Tsing Hua University, Hsinchu, Taiwan; 2 Institute of NanoEngineering and MicroSystems, National Tsing Hua University, Hsinchu, Taiwan; Brandeis University, United States of America

## Abstract

Developing methods that evaluate the cellular uptake of magnetic nanoparticles (MNPs) and nanotoxicity effects at single-cellular level are needed. In this study, magnetophoresis combining fluorescence based cytotoxicity assay was proposed to assess the viability and the single-cellular MNPs uptake simultaneously. Malignant cells (SKHep-1, HepG2, HeLa) were incubated with 10 nm anionic iron oxide nanoparticles. Prussian blue stain was performed to visualize the distribution of magnetic nanoparticles. MTT and fluorescence based assay analyzed the cytotoxicity effects of the bulk cell population and single cell, respectively. DAPI/PI stained was applied to evaluate death mechanism. The number of intracellular MNPs was found to be strongly correlated with the cell death. Significant differences between cellular MNP uptake in living and dead cells were observed. The method could be useful for future study of the nanotoxicity induced by MNPs.

## Introduction

Nanoparticles (NPs) have raised a broad interest in the field of material science or medicine [1], [2], [3], [4]. Due to the larger surface area of NPs which increase the interactions between NPs and cell membrane, the internalization of NPs by cells is effective and which is attractive for delivering drugs, probes or proteins [5], [6], [7]. Among the NPs, magnetic iron oxide NPs are considered highly biocompatible and commonly used for biomedical applications as magnetic resonance imaging (MRI) contrast agents [8], [9], to magnetically label the cells for cell separation [10] and cell sorting [11] or as heat generators for magnetic hyperthermia (MH) [12], [13].

However, cells must be labeled with large amounts of magnetic NPs (MNPs) in order to be manipulated by the above techniques, but may suffer from nanotoxicity. The main process involved in MNPs internalization is endocytosis [14], which is subjected to cell-to-cell variations [15]; therefore, nanotoxicity effects induced by MNPs should be analyzed more accurately on individual cells instead of masking by the average value of bulk measurements. Consequently, methods that assess the cellular uptake of MNPs and nanotoxicity effects at single-cell level are more reasonable than those for bulk-cell assays. Unfortunately, most of the current methods evaluate the internalized iron oxide MNP from a population of cells rather than a single cell. In addition, those techniques including Prussian blue staining [16], T2 relaxometry [17], UV/VIS spectrometry [18], [19], and atomic absorption spectroscopy (AAS) [20], [21] analyze the cells that are non-viable. Until 2002, Wilhelm *et al.* proposed magnetophoresis as a method to quantify the amounts of internalized MNPs [22]. Later in 2008, Jing *et al.* showed that magnetophoresis could not only analyze cells that remain active but also reveal the differences of uptake capacities between individual cells [23].

In regards to nanotoxicity, not only the exposure concentration/dosage but also the intrinsic properties of the nanoparticles such as surface coating or particle size, were found to be correlated with the induced nanotoxicity [24]. Studies have also observed the changes of cell morphology [25] or cytoskeleton and the increase of reactive oxygen species (ROS) [26] in response to the overloading of iron oxide NPs. From results given by Soenen *et al.*, the cytoskeleton defects have shown to be dependent on the intracellular MNP concentrations [27], [28]. The above studies about nanotoxicity, however, determined the concentration of MNPs in a statistical/bulk manner instead of single cell level; that is, nanotoxicity influenced by MNP number in an individual cell has yet to be studied. Besides, cell-to-cell distinction on MNPs uptake remains unclear. Assays based on flow-cytometry have been considered a standard method to determine the overall cell functioning of individual cell. Unfortunately, the flow-cytometry that combined the function of fluorescence analysis and quantification of MNPs has not readily been available. In this study, a simple strategy is proposed to utilize single cell magnetophoresis with fluorescence based cytotoxicity assay to examine the number of internalized MNPs and the corresponding cell viability simultaneously.

## Methods

### Cell culture and incubation with magnetic nanoparticles

Three cell lines, SKHep-1 (human liver adenocarcinoma) [29], HepG2 (hepatocellular carcinoma) [30] and HeLa (cervical adenocarcinoma) [31] were maintained in high-glucose Dulbecco's modified Eagle's medium (DMEM) supplemented with 10% fetal bovine serum, 100 U/mL penicillin and 100 mg/mL streptomycin (Gibco). The cells were cultured at 37°C in 5% CO_2_ and passaged regularly to maintain exponential growth. After seeded in 6-well culture plates to reach 80–90% confluence, the cells were changed to medium containing 10 nm anionic MNPs (iron concentration in cell medium of 260 µg/mL) (Ferrotec Corp., EMG 705) and subsequently incubated for different time periods (8, 12, 24 h). The anionic MNPs chosen in this study possess negative surface charges which is suitable for either phagocytic or non-phagocytic cell [32], [33].

### Prussian blue staining

To verify the intracellular localizations of MNPs, the cells treated with MNPs were washed with phosphate-buffered saline (PBS) for three times and fixed in 4% paraformaldehyde. Thereafter, Prussian blue stain reagent (2% potassium ferrocyanide with 6% HCl (1∶1 v/v)) were added to the cells and incubated for 10 min. Finally, cells were counterstained by nuclear fast red to visualize the cell nuclei.

### Quantification of cellular uptake of MNPs by magnetophoresis

The experimental procedure and setups for single cell magnetophoresis were similar to that was previously described [34]. Initially, a permanent magnet was adapted to the apparatus of an inverted fluorescence microscope (Olympus CKX41) which was equipped with blue, green & UV filters. The magnetic field was calibrated and the uniformity of the magnetic field was assured before conducting magnetophoresis. The magnetic cells suspended in DMEM medium were submitted to the magnetic field. In the steady state regime, the magnetic force *Fm* = *m_bead_* dB/dx (*m_bead_* is the magnetic moment of the magnetic beads and dB/dx is the magnetic field gradient) was balanced with the viscous force *F_vis_ = 6πηRν* (R is the radius of cell, η is the viscosity of the carrier liquid, *ν* is the cell velocity). The total magnetic moment of the MNPs inside a cell could be expressed as *m_bead_ = NcM_s_πD^3^/6*, where *N* is the total number of MNPs per cell, *D* is the diameter of a magnetic nanoparticle, and *c* is the ratio of the net magnetization of MNPs to their saturation magnetization *M_s_*. As the applied magnetic field was 115 mT, the magnetization corresponded to 80% of the magnetization at saturation (*M_s_* = 484 kA/m), and thus *c* could be set to 0.8. By setting the radiuses *R* of cells and the viscosity of the carrier liquid *η* as 0.013 Pas, the number of MNPs loaded by cells N could be calculated by the following equation:
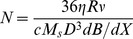
(1)


### Image analysis and equipment

The mobile cells that were attracted by the magnetic force and moved with constant speed were recorded by a high resolution cooled colored CCD camera (Micro-shot Technology Mshot). The cells were tracked for 15–20 min at one frame per second, and the image sequences were then imported into the public domain open source software, ImageJ (http://rsb.info.nih.gov/ij/, NIH, Bethesda, MD), and the displacements of individual cells were tracked via the plug-in “manual tracking” (http://rsb.info.nih.gov/ij/plugins/track/track.html, developed by F. Cordelières). To determine the cell radius, the images of cells from optical microscope was captured by CCD camera and analyzed through the built-in measurement tool of the capture software (Mshot Digital Imaging System).

### Analysis of cytotoxic effects of bulk cell population by MTT

Cells incubated with MNPs were performed MTT assay to analyze the cytotoxicity effects of bulk cell population. The reagent stock was prepared by dissolving MTT (3-(4, 5-dimethylthiazol-2-yl)-2, 5-diphenyl tetrazolium bromide), (Alfa Chemical Corp.) in PBS to reach a concentration of 5 mg/mL. Later, the stock solution was added to the cell medium in a 1∶9 (v/v) volume ratio and the cells were incubated back into the incubator for another 4 h. The supernatant in each well was then removed carefully, changed to 100% Dimethyl sulfoxide (DMSO) solution. After 10 min of mild shaking, the formazan product would be dissolved by DMSO and the absorbance (λ = 570 nm) was measured by a microplate reader. Cell viability was expressed as the percentage of the absorption of cells treated with MNPs in comparison with that of control cells.

### Single-cellular fluorescence imaging distinguishing living, apoptotic or necrotic cell

In order to visualize fluorescent signal produced by live cell, all cells were transfected with green fluorescent protein (GFP) gene [35]. Thereafter, cells were adjusted to a concentration of 10^6^ cells/100 µl PBS. For PI staining [36], 5 µL of propidium iodide (PI) (stock  = 10 µg/mL, Invitrogen) was mixed with the cell solution and incubated in the dark for 15 min. For DAPI staining, 1 µL of 4, 6-Diamidino-2-phenylindole dihydrochloride (2 µg/mL) (Sangon Biotech) was added and cells were incubated in the dark for 10 mins, washed and resuspended by medium sequentially. To define the percentage of normal, apoptotic and necrotic cell, 400 nuclei were counted in random microscopic field. The cell viability from single-cellular analysis was calculated by analyzing the fluorescence images randomly gathered from each group, and the ratio of survival to total cell number was regarded as viability (PI-positive cells with cell necrotic or late apoptotic nuclei). Right before magnetophoresis, we stained GFP-expressed cells with PI to track and analyze the fluorescence emission and the movement of each cell individually such that the information of viability and velocity for individual cell can be obtained simultaneously.

### Statistical analysis

Statistical analysis was performed using Origin 8 Pro (OriginLab Corporation, Northampton, MA). The values acquired from a population of cells were represented as mean ± standard deviation (SD). At least three independent tests were performed to reach the final results. Linear regression determined the correlation between the mean MNPs number obtained from magnetophoresis and the viability of the cells. Student's t-test was used to compare the MNP uptake between the live and dead cells in distinct cell types, and the degree of significance was given as * p<0.05; ** p<0.01; *** p<0.001.

## Results and Discussion

**Figure 1:**
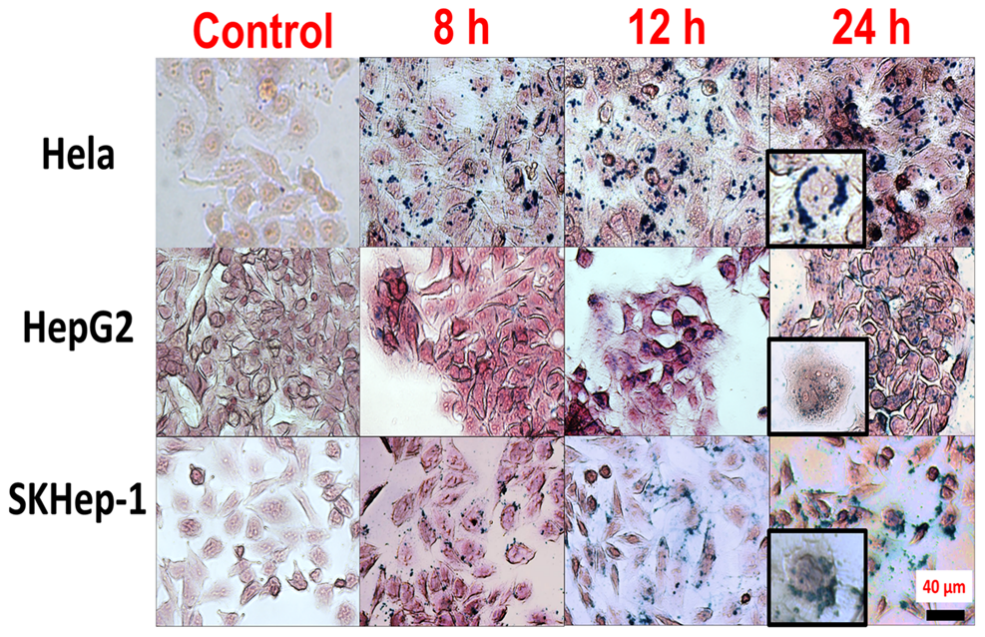
Prussian blue staining results of cancer cells internalizing magnetic nanoparticles (MNP) in a time dependent manner. Control corresponds to the cells (HepG2, SKHep-1, HeLa) that were not treated with MNPs and maintained for 24 h in parallel to the treated groups. Insets: the enlarged images of cells. Blue: MNPs; Red: nucleus. The scale bar in each image represents 40 µm.

**Figure 2:**
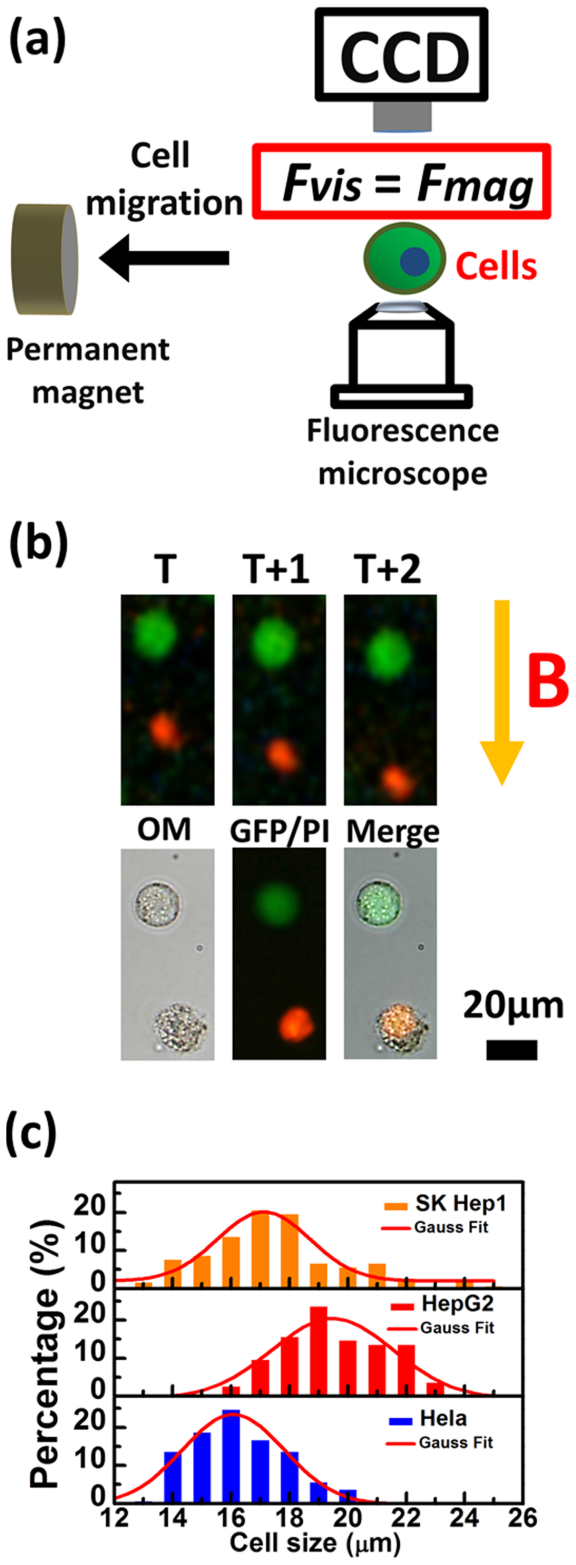
Magnetophoresis and living cell imaging. (a) Schematic representation of cell magnetophoresis. *F_mag_* and *F_vis_* indicate the magnetic force and the viscous force. (b) (Top) Consecutive magnetophoresis images of two cells move at constant speed (Bottom) Fluorescence image, optical microscope image and the merged image of the two cells. Green, live cells expressed green fluorescent protein (GFP); Red, nucleic acid stained by propidium iodide (PI). The scale bar represents 20 µm. (c) Size distributions of distinctive cells (SKHep-1, HepG2 and HeLa). The red lines are the standard normal distribution. N = 200 cells.

The cellular uptake of MNPs was evaluated by Prussian blue stain, as shown Fig. 1. Several intracellular blue-stained vesicles appeared in the cytoplasm of HepG2, HeLa and SKHep-1. Large and dense blue-stained vesicles showed as the incubation period increased, which suggested the time-dependent uptake of the anionic MNPs. In contrast, no blue deposits appeared in cells that were not treated with MNPs and maintained in parallel to the treated group (control). In Fig.2 a, the magnetic force, *F_m_ = m_bead_dB/dx* that exerted on the cells possessing magnetic moment, was balanced with the viscous force, *F_vis_ = 6πηRν* that resisted the movement of the cells caused by the motion of the cell in the medium. Therefore, the related number of intracellular MNPs could be measured as cell moved at constant speed. Fig. 2b shows the sequential micrographs of the cells whose viability was evaluated by the fluorescence probe. Since the viscous force is directly proportional to the radius of cell R, optical micrographs were obtained to analyze the cell radius (shown in Fig. 2c). The mean radiuses for HepG2, SKHep-1, and HeLa cells are 10 µm, 8.5 µm and 7.9 µm, respectively.

**Figure 3:**
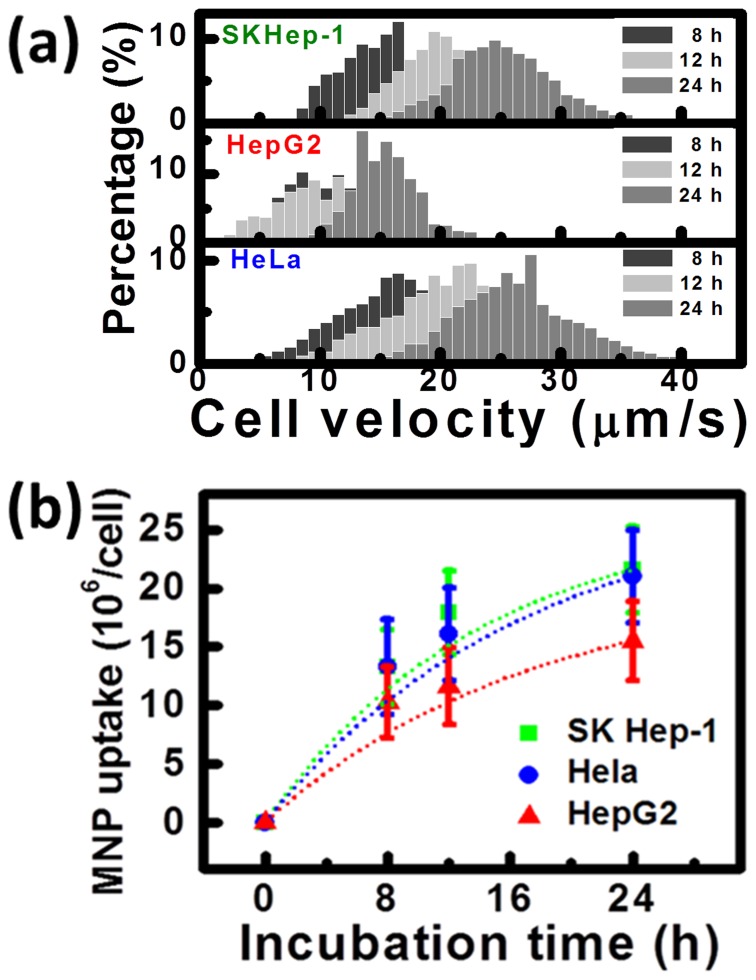
Quantitative analysis of magnetic nanoparticle (MNP) monitored as a function of the incubation time. (a) The distributions of magnetophoretic velocity of cells incubated with magnetic nanoparticles for 8, 12, 24 h. N = 1000. (b) The cell loading with MNPs after incubating for 8, 12 and 24 h. Dot lines represent the exponential fit of the data. Bars represent the standard deviation of 500 different cells.

From Fig. 3a, the magnetophoretic velocities from different cells showed Gaussian distributions which were enlarged and right shifted with the increase of time. Based on (1), the amounts of MNPs were counted from the corresponding velocity of the mobile cell and the data were plotted with the exponential fit, as shown in Fig. 3b. For SKHep-1 cells, the numbers of MNPs increased from (15.53±3.1) to (21.59±3.69)x10^6^/cell, whereas for HeLa cells, the number increased from (13.36±4.01) to (21.01±3.98)x10^6^/cell. Only a slightly increased for MNP uptake of HepG2 cells, which was from (11.17±3.35) to (15.3±3.02)x10^6^/cell. Interestingly, though SKHep-1 and HepG2 were both derived from liver, SKHep-1 uptake MNPs more effectively. According to previous research, SKHep-1 could be regarded as liver sinusoidal endothelial cells (LSEC); therefore, the better cellular uptake efficiency could be contributed to their remarkable endocytic activities [37].

**Figure 4:**
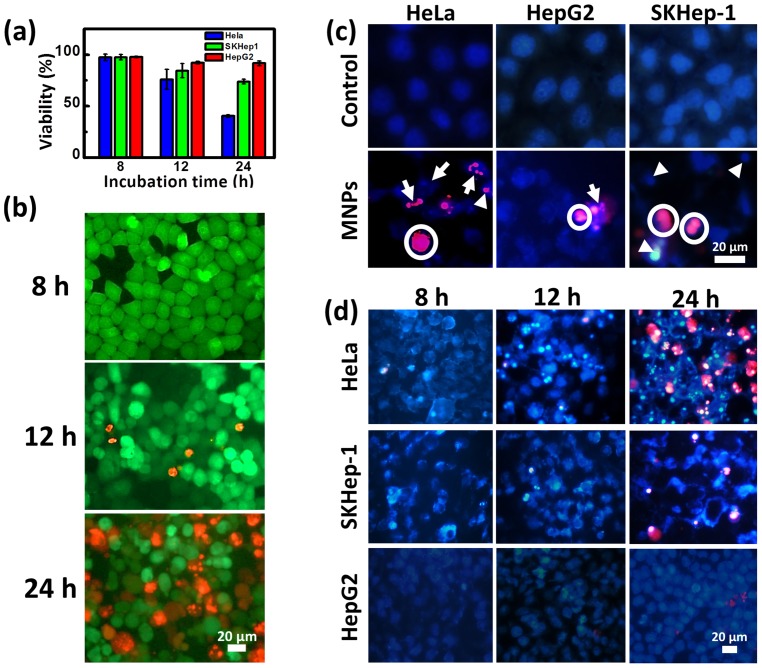
Cytotoxicity effects of single cell and bulk cell populations. (a) Cytotoxicity effects were analyzed by MTT. The data represents as the mean ±SD, and acquired from 3 independent tests. (b) The cytotoxicity effects of HeLa cells are shown by the overlay fluorescence images of GFP and PI as the MNP incubation time increased from 8 to 24 h. Green, live cells expressed green fluorescent protein (GFP); Red, dead cells stained by PI. The scale bar represents 40 µm. (c) Overlay fluorescence images of DAPI/PI stain distinguishing mechanisms of cell death (SKHep-1, HepG2 and HeLa) after treating with MNPs for 24 h. Control: cells were not treated with MNPs for 24 h in parallel to the treated groups. Apoptotic nuclei stained with DAPI showed intense blue fluorescence corresponding to chromatin condensation (indicated by arrow heads) and fragmentation (indicated by arrows). Defective cell membrane allowed PI to enter cytoplasm indicating that cells underwent late apoptosis or necrosis. Possible necrotic cell that correspond to those without clear nuclear fragment but stained with intense PI were circled. (d) Time course experiment of the DAPI/PI staining for the three kinds of cells.

Cytotoxicity effects were analyzed by bulk cell measurement, MTT as well as single-cellular fluorescence based assay. In Fig. 4a, over 90% of cell remained viable for HepG2 after 24 h of incubated with MNPs; whereas for HeLa and SKHep-1 cell, the viability decreased from 78% to 40% and 97% to 73%, respectively. MTT assay was based on the reduction of tetrazolium dye by NAD(P)H-dependent oxidoreductase enzymes in the cytosolic compartment of the cell [38]. The decrease of cell viability may be caused by the interaction between MNPs and the subcellular membrane molecule and affected metabolism [24], [39]. Researches have suggested that cells exposed to iron oxide NPs may undergo apoptosis [40]; therefore, DAPI/PI stained was applied to evaluate death mechanism. Apoptotic cells was characterized by abnormal, condensed or fragment chromosome stained by DAPI dye; while for PI dye, it penetrated dead cell membranes to bind to the intracellular DNA, and identified both late-apoptotic and necrotic cells as non-viable cells. In Fig. 4b, the number of non-viable HeLa cells stained by PI increased with the incubation time, which was similar to the result obtained from MTT. In Fig. 4c, the DAPI/PI staining indicated that cells underwent late apoptosis or necrosis after treating with MNPs for 24 h. As high as 61% cells were in necrosis/late-apoptosis for HeLa, as illustrated in Table 1. To prove that cells were traversing through early apoptosis before reaching late apoptosis/necrosis, time course experiment was performed (shown in Fig. 4d). After the initially 8 h of incubation time, condensed chromatin structure with fragmented DNA started to appear inside cells but few were stained by PI, which could be characterized as early-apoptosis [41], [42]. As incubation time increased from 12 to 24 h, defects occurred in the cell membrane of apoptotic cells and allowed PI to enter the cytoplasm.

**Table 1:** The mean percentage of normal, apoptotic and necrotic cell stained with DAPI/PI.

Cell	% Normal*			% Apoptosis†			% Late apoptosis/necrotic‡		
	8 h	12 h	24 h	8 h	12 h	24 h	8 h	12 h	24 h
**HeLa**	87.91±2.94	34.5±3.04	19±5.03	10.25±2.87	43±2.25	20±1.5	1.84±0.87	22.5±0.8	61±3.6
**SKHep-1**	87.63±1.4	63.5±3.22	65.8±3	10.75±1.7	22±3.1	9.6±1.15	1.61±0.33	14.5±0.92	24.6±5.13
**HepG2**	96.09±0.88	93±2	90.1±1.1	3.51±0.85	1±0.5	2.8±0.4	0.4±0.1	6±2	7.1±1.15

*With homogenous DAPI.

† Condensed or fragmented DNA stained by DAPI.

‡ Defective cell membrane stained by PI.

**Figure 5:**
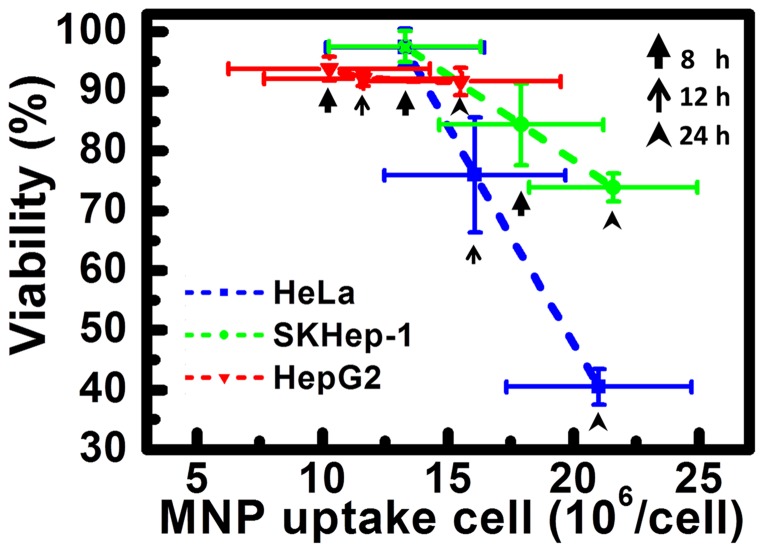
The correlation between the number of internalized MNPs and cell viability. The three data points for each cell type represent the three incubation times (8, 12, 24 h) with MNPs. The ratio of survival to total cell number was regarded as viability (PI-positive cells with cell necrosis or late apoptotic nuclei were regarded as dead cells). The error bars represent the standard deviations (SD) obtained from three independent replicates. The correlation between the mean MNPs number obtained from magnetophoresis and the viability of the cells was determined by linear regression.

**Figure 6:**
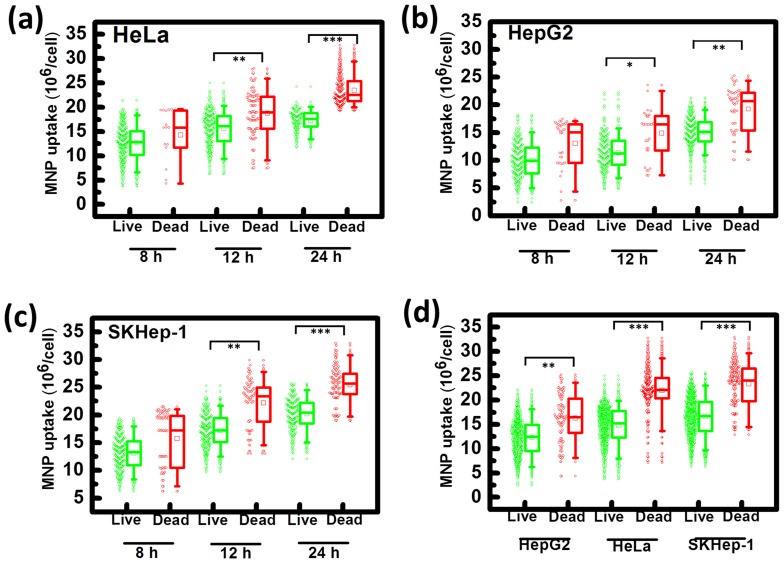
The variability of the number of intracellular MNPs obtained from live (green) and dead (red) cell. (a)∼(c) are box plots of HeLa, HepG2 and SKHep-1, respectively. For each cell type, 500 cells that were treated with MNPs were analyzed at different co-culture times (8, 12, 24 h) to obtain the uptaken MNP number and viability of an individual cell. (d) is the data collection of all 1500 cells with different co-culture times (8, 12, 24 h) for each cell type. The dead/live cells were discriminated by the fluorescence images in the magnetophoresis experiment. The box represents the quartile distribution (25–75%) range. The median is indicated by a horizontal line and the mean is indicated by an open square inside the box. The whiskers mark 5–95% range of the distribution, and the outlier samples are indicated outside of this range. The scatter plots indicate the uptaken MNP number obtained from individual cell. Statistical significance between dead and live cells was determined using a student's t-test. The degree of significance was given as *: p<0.05; **: p<0.01; ***: p<0.001.

In Fig 5, the viability from fluorescence images and the corresponding number of internalized MNPs showed good linearity (R^2^>0.9999) for both SKHep-1 and HeLa cells, which suggested nanotoxicity should have strong correlation with the number of intracellular MNPs. In Fig. 6, the MNP number obtained individually from dead/live cells were presented by box plot and cell-to-cell distinctions on MNPs uptake were appeared. Significantly differences between cellular MNP uptake in living and dead cells were observed after 12 h of incubation time (p<0.05 for HepG2; p<0.01 for SKHep-1, HeLa); as incubation time reached 24 h, the differences became more distinct for HeLa and SKHep-1(p<0.001) than HepG2 cell. Research suggested that there might be certain threshold for internalized NPs to exert effects on cell physiology [27]. Data of dead and live cells in the three incubation time (8, 12 and24 h) were gathered to gain a possible range of MNP uptake that triggered cell death (shown in Fig. 6d). The comparison between the number of uptaken MNP in viable and non-viable cell were expressed as follow: SKHep-1((16±4.08) vs. (23±4.49)x10^6^/cell), HepG2 ((12.28±3.63) vs. (16.27±4.86)x10^6^/cell) and HeLa ((14.7±3.71) vs. (21.98±4.27)x10^6^/cell). Since the number of uptaken MNP is highly dependent on the endocytic activities, the number that obtained from the viable/non-viable cells of SKHep-1 and HeLa cell are much higher than that from HepG2 cell. Whereas, for the cells exhibited higher/lower activity or in phase between early-apoptosis to late apoptosis, the number of uptaken MNP would differ from the average and occurred as subpopulation in live/dead cell.

In our study, we proposed magnetophoresis combining fluorescence based cytotoxicity assay to evaluate the viability and the cellular MNPs uptake simultaneously at single cell level such that the influence of MNP number on cell viability of individual cells analyzed. Besides, using this method, endocytosis capacities of MNPs were analyzed individually and they exhibited cell-to-cell variations. So far, most of the previous studies estimated the concentration of iron oxide NPs by analyzing the constituent iron atoms or ions (Fe^2+^, Fe^3+^) dissociated from iron oxide NPs, whereas the magnetophoresis method used in the present study can directly derive the number of iron oxide NPs in their solid form.

## Conclusions

In summary, magnetophoresis with fluorescence based cytotoxicity assay was proposed to evaluate the viability and MNPs loading simultaneously. By the method, endocytosis capacities of MNPs were analyzed individually to show cell-to-cell variations. The number of intracellular MNPs quantified by magnetophoresis was found to be strongly correlated with the viability and the differences between cellular MNP uptake in living and dead cells were distinctive. The method could be useful for future study of nanotoxicicy induced by MNPs.
